# Distinct Expression Patterns of miR-21-5p and miR-21-3p in Oral Epithelial Dysplasia and Early Oral Squamous Cell Carcinoma

**DOI:** 10.3390/cancers18193116

**Published:** 2026-09-25

**Authors:** Miloš Trajković, Dragan Krasić, Marija Vukelić-Nikolić, Predrag Radović, Tatjana Jevtović-Stoimenov, Simona Stojanović, Nikola Živković, Miloš Stojanović, Nikola Krstić

**Affiliations:** 1 Department of Maxillofacial Surgery, Clinic for Dental Medicine, Faculty of Medicine, University of Niš, 18108 Niš, Serbia; drkrasicnis@gmail.com (D.K.);; 2 Department for Biology and Human Genetics, Faculty of Medicine, University of Niš, 18108 Niš, Serbia; 3 Department of Biochemistry, Faculty of Medicine, University of Niš, 18108 Niš, Serbia; tatjana.jevtovic.stoimenov@medfak.ni.ac.rs; 4 Department of Oral Surgery, Clinic for Dental Medicine, Faculty of Medicine, University of Niš, 18108 Niš, Serbia; 5 Department of Pathology, Institute of Pathological Anatomy, Clinical Center, Faculty of Medicine, University of Niš, 18108 Niš, Serbia; 6 Laboratory for Functional Genomics and Proteomics, Faculty of Medicine, University of Niš, 18108 Niš, Serbia

**Keywords:** oral leukoplakia, epithelial dysplasia, oral squamous cell carcinoma, miR-21-5p, miR-21-3p, malignant transformation, biomarker

## Abstract

**Background/Objectives**: Oral leukoplakia (OL) is a common oral potentially malignant disorder, but prediction of malignant progression remains challenging. This study investigated miR-21-5p and miR-21-3p expression in healthy oral mucosa, OL with epithelial dysplasia, and early oral squamous cell carcinoma (OSCC), together with clinicopathological associations. **Methods**: Sixty participants were divided into three groups: exposure-free healthy controls (*n* = 20), histopathologically confirmed OL with epithelial dysplasia (*n* = 20), and early OSCC (pT1–2N0M0; *n* = 20). Expression was quantified by RT-qPCR and evaluated using exploratory correlation, regression, Welch’s ANOVA, and Games–Howell comparisons. **Results**: miR-21-5p differed significantly among groups (*p* < 0.001). Expression was lower in OL than in healthy controls and early OSCC (both *p* < 0.001), whereas early OSCC and controls did not differ (*p* = 0.997). miR-21-5p was inversely associated with dysplasia grade (Spearman’s ρ = −0.54, *p* = 0.013; unstandardized regression coefficient B = −0.646, *p* = 0.017). miR-21-3p was numerically higher in early OSCC, but its overall group difference was not significant (*p* = 0.051). **Conclusions**: The two mature miR-21 strands showed distinct, non-linear expression patterns across the three groups. These findings are preliminary and hypothesis-generating; the cross-sectional design does not establish predictive or prognostic value, and validation in larger longitudinal cohorts is required. The unexpected miR-21-5p pattern remains unexplained and requires independent validation.

## Simple Summary

Oral epithelial dysplasia can occur in oral leukoplakia, but it remains difficult to determine which lesions will progress toward oral cancer. We compared two mature forms of microRNA-21, miR-21-5p and miR-21-3p, in healthy oral mucosa, oral leukoplakia with epithelial dysplasia, and early oral squamous cell carcinoma. The two strands showed different expression patterns. miR-21-5p was markedly reduced in dysplastic oral leukoplakia and was associated with the severity of epithelial dysplasia, whereas miR-21-3p was numerically higher in early oral cancer without reaching statistical significance. These preliminary cross-sectional findings indicate group-associated molecular differences and support separate evaluation of the two strands in larger longitudinal studies; they do not establish predictive or prognostic utility.

## 1. Introduction

Oral squamous cell carcinoma (OSCC) is the most common malignant neoplasm of the oral cavity. It is characterized by locally aggressive growth and considerable metastatic potential. Five-year overall survival is approximately 63%, with reported survival of about 83% in early-stage disease and 38% in advanced-stage disease [1]. OSCC develops through a complex interplay of genetic and epigenetic alterations associated with environmental exposures and other biological factors. Tobacco and alcohol consumption are the principal established lifestyle risk factors, whereas high-risk human papillomavirus (HPV) is a major etiological factor for oropharyngeal squamous cell carcinoma but contributes only to a minority of oral cavity SCCs [2,3]. Oral potentially malignant disorders (OPMDs) represent clinically identifiable conditions associated with an increased risk of subsequent OSCC development. These exposures and precursor conditions may promote the accumulation of molecular alterations that disrupt cell-cycle regulation, proliferation, differentiation, and apoptosis, facilitating progression from normal epithelium through epithelial dysplasia to invasive carcinoma. Despite substantial advances in surgical and oncological treatment [4,5], the prognosis of patients with advanced-stage disease remains poor. Therefore, early detection and appropriate management of OPMDs remain central strategies for reducing the burden of OSCC.

Oral leukoplakia (OL) is the most common oral potentially malignant disorder and accounts for a substantial proportion of malignant transformations among OPMDs. Clinically, OL is classified into homogeneous and non-homogeneous forms, with non-homogeneous lesions carrying a substantially higher risk of malignant transformation. A recent comprehensive meta-analysis estimated a pooled malignant transformation proportion of 6.64% (95% CI, 5.21–8.21), while also demonstrating substantial heterogeneity among studies and a higher transformation risk in non-homogeneous lesions and lesions with epithelial dysplasia [6]. Management is based on histopathological assessment, epithelial dysplasia grade, lesion location, clinical characteristics, and elimination of individual risk factors. The histological grade of epithelial dysplasia is among the most important indicators of malignant transformation risk. Lesions with intermediate- and high-grade dysplasia are commonly managed by surgical excision, whereas lower-risk lesions require careful long-term clinical surveillance [7,8,9,10]. However, the biological behavior of OL does not always correspond to the histological grade of dysplasia; consequently, histopathological assessment alone cannot reliably identify which lesions will undergo malignant transformation.

Because conventional clinical and histopathological parameters have limited predictive value, increasing attention has been directed toward molecular biomarkers that may improve individual risk assessment and therapeutic decision-making. MicroRNAs (miRNAs) have attracted particular interest. These small non-coding RNAs are approximately 18–24 nucleotides in length and regulate gene expression at the post-transcriptional level. They participate in numerous biological processes, including proliferation, differentiation, apoptosis, angiogenesis, and epithelial–mesenchymal transition, and have an important role in carcinogenesis [11,12,13].

Among these molecules, miR-21 is one of the most frequently dysregulated miRNAs in solid malignancies, including OSCC. Increased expression of total miR-21 has been associated with aggressive tumor behavior, local recurrence, metastasis, and poorer prognosis. Two mature strands, miR-21-5p and miR-21-3p, are generated from the same precursor and may exert distinct biological effects. Increased miR-21-5p expression has been associated with tumor cell proliferation, invasion, therapeutic resistance, and tumor progression, whereas the biological role of miR-21-3p across different grades of epithelial dysplasia, OL, and early oral carcinogenesis remains incompletely understood [14,15,16]. Although total miR-21 expression has been extensively investigated in OSCC, data on the individual expression patterns of miR-21-5p and miR-21-3p during early oral carcinogenesis remain limited. Their associations with the histopathological characteristics of OL also require further investigation. A better understanding of these strand-specific expression patterns may contribute to the identification of molecular markers associated with early oral carcinogenesis and may complement conventional clinicopathological assessment.

The aim of this study was to compare miR-21-5p and miR-21-3p expression in healthy oral mucosa, OL with epithelial dysplasia, and early-stage OSCC. We also evaluated their associations with clinical and histopathological characteristics, with particular emphasis on epithelial dysplasia grade, and explored strand-specific expression differences across these cross-sectional disease groups.

## 2. Materials and Methods

### 2.1. Study Design and Ethical Approval

This prospective observational study was conducted between 2023 and 2026 through a multidisciplinary collaboration involving the Department of Maxillofacial Surgery, the Center for Pathology and Pathological Anatomy, and the Department of Microbiology at the University Clinical Center Niš, together with the Scientific Research Center For Biomedicine and Laboratory for Medical Genetics, Faculty of Medicine, University of Niš, Serbia.

The study protocol was approved by the Ethics Committee of the Clinic of Dental Medicine (Approval No. 14/14-2019-4EO), and the Ethics Committee of the Faculty of Medicine, University of Niš (Approval No. 12-16502/2-1). All procedures were performed in accordance with the Declaration of Helsinki and its subsequent amendments.

Before enrollment, all participants received detailed information regarding the study objectives, surgical procedures, histopathological and molecular analyses, and data confidentiality. Written informed consent was obtained from all participants prior to inclusion in the study.

### 2.2. Study Population

A total of 60 participants were enrolled and allocated into three study groups comprising 20 subjects each:

− Healthy oral mucosa (control group);
− Oral leukoplakia (OL);
− Early oral squamous cell carcinoma (OSCC).

Patients were consecutively recruited during the study period according to predefined inclusion and exclusion criteria.

### 2.3. Healthy Control Group

The control group consisted of twenty individuals with clinically healthy oral mucosa.

The exclusion criteria included:

− Chronic inflammatory lesions of the oral mucosa;
− Oral potentially malignant disorders (OPMDs);
− Ulcerative, verrucous, or exophytic lesions suggestive of oral malignancy;
− Current tobacco or alcohol consumption;
− Previous diagnosis or treatment of OPMDs or oral cancer.

The control group was restricted to individuals without current tobacco or alcohol exposure in order to minimize potential confounding effects of these established environmental exposures on miRNA expression and to provide a more biologically homogeneous reference group. A standardized excisional biopsy of clinically healthy buccal mucosa was performed for tissue sampling. Buccal mucosa was selected as the standardized sampling site because of its easy surgical accessibility, suitability for obtaining an adequate tissue specimen with minimal morbidity, and the straightforward management of potential local complications. This standardized approach was intended to reduce variability related to sampling site and modifiable environmental exposures within the control group. Tissue specimens were divided into two portions. One fragment was submitted for routine histopathological examination to confirm the absence of pathological alterations, whereas the second fragment was immediately stored at −80 °C until molecular analysis.

### 2.4. Oral Leukoplakia Group

The second study group consisted exclusively of patients with clinically diagnosed oral leukoplakia and histopathologically confirmed epithelial dysplasia. Patients with oral leukoplakia without epithelial dysplasia were not included in the study.

The exclusion criteria were:

− Multiple leukoplakia lesions;
− Proliferative verrucous leukoplakia (PVL);
− Coexistence of another oral potentially malignant disorder;
− Previous diagnosis or treatment of oral leukoplakia;
− Previous history of oral malignancy;
− Clinical suspicion of invasive carcinoma confirmed by biopsy.

Patients with proliferative verrucous leukoplakia (PVL) were excluded because PVL represents a distinct clinicopathological entity with different biological behavior and a substantially higher risk of malignant transformation.

Following excisional biopsy, each tissue specimen was divided into two portions. One fragment underwent routine histopathological examination for confirmation of oral leukoplakia and grading of epithelial dysplasia, whereas the remaining tissue was stored at −80 °C until molecular analysis.

### 2.5. Early Oral Squamous Cell Carcinoma Group

The third study group consisted of patients with oral squamous cell carcinoma confirmed by incisional biopsy.

Preoperative staging was established by clinical examination and radiological imaging. Only patients with clinically staged cT1–2N0M0 disease who underwent primary surgical treatment and were subsequently confirmed as pathological stage pT1–2N0M0 on final histopathological examination were included.

The exclusion criteria included:

− Advanced tumors (cT3–cT4);
− Recurrent oral squamous cell carcinoma;
− Distant metastases;
− Initially inoperable tumors requiring neoadjuvant therapy;
− Disease progression before surgery;
− Contraindications to surgery under general anesthesia.

Following tumor excision, tissue specimens were divided into two portions. One fragment was submitted for routine histopathological examination, whereas the remaining tissue was immediately stored at −80 °C until molecular analysis.

### 2.6. Histopathological Evaluation

Histopathological examinations were performed at the Center for Pathology and Pathological Anatomy, University Clinical Center Niš.

Tissue specimens were fixed in 10% neutral buffered formalin for at least 24 h, followed by routine tissue processing and paraffin embedding. Serial sections (5 μm thick) were then prepared and stained with hematoxylin and eosin (H&E). Formalin, paraffin, hematoxylin, and eosin were obtained from Merck/Sigma-Aldrich (Merck KGaA, Darmstadt, Germany).

All histopathological specimens were evaluated by an experienced oral pathologist before molecular analysis.

Histopathological evaluation confirmed normal oral mucosa in the control group, oral leukoplakia with epithelial dysplasia in the second group, and oral squamous cell carcinoma in the third group. Epithelial dysplasia was graded by the oral pathologist using a three-tier system as low-, intermediate-, or high-grade. For presentation and comparison with the current WHO three-tier classification, these categories were harmonized as mild, moderate, and severe dysplasia, respectively. The WHO classification retains a three-tier grading system comprising mild, moderate, and severe oral epithelial dysplasia [17].

### 2.7. Microbiological Analysis

Prior to biopsy, oral swab specimens were collected from the oral cavity for microbiological analysis. The samples were cultured on Sabouraud dextrose agar (Torlak Institute, Belgrade, Serbia) and processed according to the routine procedures of the Department of Microbiology. *Candida albicans* was identified using standard microbiological methods routinely employed in the laboratory. The presence or absence of *Candida albicans* colonization was recorded and included as a clinicopathological variable in the subsequent statistical analyses.

### 2.8. RNA Isolation and Reverse Transcription

Total RNA, including microRNA, was isolated from frozen tissue samples using the miRNeasy Mini Kit (Qiagen, Hilden, Germany) according to the manufacturer’s instructions.

For each sample, RNA concentration and purity were determined spectrophotometrically using a NanoDrop™ One Spectrophotometer (Thermo Fisher Scientific, Waltham, MA, USA). All samples were diluted with RNase/DNase-free water to a final concentration of 5 ng/μL before reverse transcription.

Reverse transcription was performed using the miRCURY LNA RT Kit (Qiagen, Hilden, Germany) in a Mastercycler ep gradient S (Eppendorf, Hamburg, Germany). The thermal profile consisted of 42 °C for 60 min, followed by 95 °C for 5 min, and cooling to 4 °C.

The resulting cDNA was diluted 1:30 before quantitative real-time PCR analysis.

### 2.9. Quantitative Real-Time PCR

Quantitative real-time PCR (RT-qPCR) was performed on a Stratagene Mx3005P system (Agilent Technologies, Santa Clara, CA, USA) with MxPro QPCR software (V4.10d) (Agilent Technologies, Santa Clara, CA, USA), using KAPA SYBR^®^ FAST Master Mix Universal (KAPA Biosystems, Wilmington, MA, USA) together with commercially available miRCURY LNA miRNA PCR Assays (Qiagen) specific for hsa-miR-21-5p, hsa-miR-21-3p, and *SNORD48*, which served as the endogenous reference gene. The complete list of primer assays used in this study is presented in Table 1.

PCR amplification was performed using the following cycling conditions: initial denaturation at 95 °C for 2 min, followed by 40 amplification cycles consisting of 95 °C for 10 s and 56 °C for 60 s. Amplification specificity was verified by melt curve analysis performed at 95 °C for 1 min, 60 °C for 30 s, and 95 °C for 30 s. All RT-qPCR reactions were performed in duplicate, and the mean Ct value was used for subsequent analysis.

### 2.10. Relative miRNA Expression Analysis

Relative expression levels of miR-21-5p and miR-21-3p were calculated using the comparative 2^−ΔΔCt^ method. *SNORD48* served as the endogenous reference gene, and the mean ΔCt of all healthy oral mucosa was used as the calibrator. Relative expression was initially expressed as fold change (FC) according to the following equation:

Fold Change (FC) = 2^−ΔΔCt^

For statistical analysis and graphical presentation, relative expression values were log_2_-transformed and expressed as log_2_ fold change (log_2_FC).

### 2.11. Statistical Analysis

Data preparation was performed using Microsoft Excel (Microsoft 365; Microsoft Corporation, Redmond, WA, USA), whereas all statistical analyses were carried out using IBM SPSS Statistics version 27.0 (IBM Corp., Armonk, NY, USA).

Categorical variables are presented as frequencies and percentages, whereas continuous variables are expressed as mean ± standard deviation (SD).

The normality of data distribution was assessed using the Shapiro–Wilk test.

Exploratory associations between clinicopathological variables and miR-21 expression within the OL group were evaluated using Spearman’s rank correlation. Age at specimen collection was analyzed as a continuous variable; sex (female = 0, male = 1), alcohol consumption (no = 0, yes = 1), smoking (no = 0, yes = 1), combined exposure (no = 0, yes = 1), *C. albicans* colonization (absent = 0, present = 1), and clinical type (homogeneous = 0, non-homogeneous = 1) were coded dichotomously; and epithelial dysplasia was coded ordinally (low = 1, intermediate = 2, high = 3). Anatomical site was summarized descriptively and was not assigned an ordinal score because the categories have no inherent order and several cells were small. Given the limited sample size and exploratory use of categorical variables, these analyses were interpreted cautiously. No multiplicity adjustment was applied to these exploratory clinicopathological correlations; their *p* values are therefore nominal and should be interpreted as hypothesis-generating. Linear regression was additionally used to evaluate the association between dysplasia grade and each mature strand. Within each study group, the association between miR-21-5p and miR-21-3p expression was evaluated using Spearman’s rank correlation.

Differences in miR-21-3p and miR-21-5p expression among healthy controls, OL, and early OSCC were assessed using one-way analysis of variance (ANOVA). When the assumption of homogeneity of variances was violated, Welch’s ANOVA was used. Where an overall difference was statistically significant, pairwise comparisons were performed using the Games–Howell post hoc test.

A two-sided *p*-value < 0.05 was considered statistically significant.

## 3. Results

### 3.1. Demographic and Clinicopathological Characteristics of the Study Groups

A total of 60 participants were included, with 20 in each group. Mean age at specimen collection was 55.5 ± 10.21 years in healthy controls, 60.1 ± 11.93 years in OL, and 62.5 ± 11.23 years in early OSCC. Male participants represented 55%, 55%, and 65% of the respective groups. All controls were non-smokers, reported no alcohol consumption, and provided buccal-mucosa samples. Smoking was reported by 30% of participants with OL and 65% with early OSCC; alcohol consumption was reported by 65% and 40%, respectively; and combined exposure was present in 25% and 40%, respectively.

Sampling sites and OL-specific characteristics are summarized with the demographic and exposure data in Table 2. OL most often involved the floor of the mouth (40%) or ventral tongue (30%), whereas early OSCC most often involved the tongue (60%). Within the OL group, low-, intermediate-, and high-grade epithelial dysplasia occurred in 25%, 40%, and 35% of cases, respectively. *C. albicans* colonization was present in 65%, and 60% of lesions were non-homogeneous.

### 3.2. Association Between Clinicopathological Variables and miR-21 Expression

Spearman correlation analysis demonstrated no statistically significant association between any examined variable and miR-21-3p expression. miR-21-5p expression was inversely associated with epithelial dysplasia grade (ρ = −0.54, *p* = 0.013), indicating lower expression at higher grades. *C. albicans* colonization was not significantly associated with miR-21-3p (ρ = −0.21, *p* = 0.376) and miR-21-5p (ρ = −0.39, *p* = 0.088). No significant associations were observed between miR-21-5p and age, sex, smoking, alcohol consumption, combined exposure, or clinical type. The exploratory correlation results are shown in Figure 1.

### 3.3. Linear Regression Analysis

Linear regression analysis demonstrated a significant inverse association between epithelial dysplasia grade and miR-21-5p expression (unstandardized regression coefficient B = −0.646, 95% CI −1.162 to −0.131, *p* = 0.017) (Table 3). No statistically significant association between epithelial dysplasia grade and miR-21-3p expression was identified.

The negative regression coefficient indicates that lower epithelial dysplasia grades were associated with higher miR-21-5p expression within the OL group.

### 3.4. Differential Expression of miR-21 Mature Strands Among the Study Groups

Relative expression levels of miR-21-3p and miR-21-5p across the study groups are summarized in Table 4 and illustrated in Figure 2.

Because the assumption of homogeneity of variances was violated, Welch’s ANOVA was applied. The between-group difference in miR-21-3p expression did not reach statistical significance (*p* = 0.051); therefore, post hoc pairwise comparisons were not performed.

For miR-21-5p, Welch’s ANOVA demonstrated a statistically significant overall difference among groups (*p* < 0.001). Games–Howell analysis showed lower expression in OL than in healthy controls (mean difference, −4.25 log_2_FC; 95% CI, −5.07 to −3.44; *p* < 0.001) and early OSCC (mean difference, −4.31; 95% CI, −6.19 to −2.42; *p* < 0.001). Early OSCC and healthy controls did not differ (mean difference, 0.06; 95% CI, −1.85 to 1.96; *p* = 0.997).

### 3.5. Relationship Between miR-21-5p and miR-21-3p Expression Within Study Groups

Within-group analysis demonstrated a moderate positive association between miR-21-3p and miR-21-5p expression in healthy controls (Spearman’s ρ = 0.50, *p* = 0.025) and a strong positive association in OL (Spearman’s ρ = 0.71, *p* < 0.001). No association was observed in early OSCC (Spearman’s ρ = −0.04, *p* = 0.880) (Figure 3).

## 4. Discussion

Oral leukoplakia (OL) is the most common oral potentially malignant disorder (OPMD) and one of the most important precursor lesions of oral squamous cell carcinoma (OSCC). Despite substantial progress in understanding the molecular mechanisms of oral carcinogenesis, assessment of an individual lesion’s risk of malignant transformation remains a major clinical challenge. The biological behavior of OL reflects a complex interaction among patient characteristics, established risk factors, clinical and histopathological features of the lesion, and molecular alterations. Risk assessment therefore requires integration of multiple parameters rather than reliance on a single isolated variable [2,3,6,7].

Mean age at specimen collection was 55.5 years in healthy controls, 60.1 years in OL, and 62.5 years in early OSCC; the proportions of male participants were 55%, 55%, and 65%, respectively. Within the OL group, neither age nor sex was significantly associated with either mature strand. These variables therefore did not explain the within-group variability in miR-21 expression, although the modest between-group age difference remains relevant when interpreting group contrasts.

The exposure distributions differed by design and disease group. Healthy controls were selected to have no current tobacco or alcohol exposure, whereas smoking was reported by 30% of the OL group and 65% of the early OSCC group, and alcohol consumption by 65% and 40%, respectively. Combined exposure occurred in 25% of OL and 40% of early OSCC. Within OL, neither exposure was significantly associated with either strand, but the small sample provides limited power to exclude modest effects. Experimental tobacco exposure can alter miRNA profiles in oral keratinocytes [18], and an HNSCC cohort reported higher miR-21 expression among alcohol consumers [19]. Consequently, the between-group expression differences cannot be attributed solely to lesion status; residual confounding from the deliberately unmatched exposure distributions remains possible.

An important histopathological feature of our cohort was the distribution of epithelial dysplasia: intermediate-grade dysplasia was present in 40% of patients and high-grade dysplasia in 35%. Previous studies have shown that the risk of malignant transformation increases with increasing severity of epithelial dysplasia [6,20,21]. Nevertheless, histopathological assessment has recognized limitations. Clinical management cannot be based solely on dysplasia grade and should also consider the clinical appearance, location, and other characteristics of the lesion [8]. Moreover, malignant transformation can occur in lesions without marked epithelial dysplasia [10], further supporting the investigation of molecular markers that may complement conventional histopathological evaluation.

Non-homogeneous leukoplakia was the predominant clinical subtype, accounting for 60% of lesions. Previous studies have consistently associated non-homogeneous lesions with a higher risk of malignant transformation than homogeneous leukoplakia [6,20,22]. In our study, however, clinical subtype was not significantly associated with miR-21-3p or miR-21-5p expression. The lack of a detectable association may partly reflect the limited sample size, but it does not diminish the established clinical relevance of non-homogeneous lesions or the need for timely biopsy and long-term surveillance.

The floor of the mouth (40%) and ventral tongue (30%) were the most frequent OL sites, whereas 60% of early OSCC specimens arose from the tongue. These anatomical regions are clinically relevant to oral carcinogenesis [22,23]. Because anatomical site is nominal and site-specific subgroups were small, we did not impose an ordinal coding or perform an inferential site analysis. The difference between standardized buccal-mucosa sampling in controls and the diverse lesion sites in OL and OSCC should therefore be considered a potential source of tissue heterogeneity.

*C. albicans* colonization was detected in 65% of patients with OL. McCullough et al. reported an association between oral yeast carriage and epithelial dysplasia [24], whereas Ramirez-Garcia et al. described mechanisms through which *C. albicans* might contribute to carcinogenesis, including acetaldehyde production, oxidative stress, chronic inflammation, and modulation of the local immune response [25]. In our study, *C. albicans* colonization was not significantly associated with miR-21-3p (ρ = −0.21, *p* = 0.376) or miR-21-5p (ρ = −0.39, *p* = 0.088). The latter estimate may merit examination in a larger sample, but the present data do not support *C. albicans* as an independent molecular marker.

Overall, none of the examined clinical factors showed a statistically significant association with miR-21-3p or miR-21-5p expression, whereas epithelial dysplasia grade was significantly associated with miR-21-5p expression. Long-term studies indicate that malignant transformation of OL cannot be reliably predicted using a single clinical or histopathological parameter [20,21,22]. These observations support current efforts to integrate clinical, histopathological, and molecular information for more refined risk stratification.

In this context, miRNAs represent a particularly attractive class of candidate biomarkers because of their relative stability and their ability to regulate multiple genes involved in key processes of tumor biology. Most previous studies in OSCC have focused on total miR-21 expression or on the more frequently investigated mature strand miR-21-5p, whereas data on miR-21-3p remain comparatively limited. Simultaneous analysis of miR-21-5p and miR-21-3p in healthy oral mucosa, OL, and early OSCC may therefore provide additional insight into their potential roles at different stages of oral carcinogenesis.

miR-21 is one of the most extensively studied oncogenic miRNAs. Chang et al. demonstrated increased miR-21 expression in HNSCC, while experimental inhibition of miR-21 reduced proliferation and increased apoptosis [26]. Its pro-oncogenic effects have been linked, among other mechanisms, to regulation of the tumor-suppressor gene *PDCD4* [27,28]. miR-21 has also been reported to regulate RECK and TIMP3, thereby influencing matrix metalloproteinase activity and invasive tumor-cell behavior [29].

In our study, miR-21-3p expression was similar in healthy oral mucosa and OL but numerically higher in early OSCC (log_2_FC = 1.47). However, after application of Welch’s ANOVA, the between-group difference did not reach the conventional threshold for statistical significance (*p* = 0.051). This finding should therefore be interpreted as a trend toward higher miR-21-3p expression in early OSCC rather than evidence of statistically significant upregulation. The observed pattern may indicate more pronounced alterations in this mature strand after acquisition of a malignant phenotype, but this interpretation requires confirmation in larger cohorts.

Data on the specific role of miR-21-3p in OSCC remain limited. Tseng et al. analyzed miR-21-3p in 95 OSCC specimens and reported higher expression in tumor tissue than in corresponding normal tissue. Higher miR-21-3p levels were associated with N classification, while inhibition of miR-21-3p reduced the invasive capacity of OSCC cells [30]. Our findings are partly consistent with these observations because the highest numerical miR-21-3p expression was observed in the early OSCC group. Nevertheless, the absence of statistical significance in our cohort requires cautious interpretation. The relatively small sample size, restriction of the OSCC cohort to pT1–2N0M0 tumors, and biological heterogeneity of OSCC may have contributed to this result. Other miRNA expression-profiling studies in OSCC have likewise reported higher miR-21-3p expression in tumor than in normal tissue [31].

In contrast to miR-21-3p, miR-21-5p showed a pronounced and statistically supported expression pattern. Its expression was significantly lower in OL than in healthy oral mucosa (log_2_FC = −4.25; *p* < 0.001) and early OSCC (*p* < 0.001), whereas early OSCC and healthy controls did not differ significantly (*p* = 0.997). This result does not support a simple linear model in which miR-21-5p expression progressively increases from normal epithelium through OL to invasive carcinoma. The observed group differences should not be interpreted as evidence of a defined temporal or stage-specific regulatory trajectory.

The absence of increased miR-21-5p expression in our early OSCC group differs from much of the published literature. Increased expression of total miR-21 or specifically miR-21-5p has been reported in multiple HNSCC and OSCC cohorts [26,32,33,34]. The reason for this discrepancy cannot be determined from the present study. Methodological and biological heterogeneity among studies may be relevant, including disease stage, histopathological tumor characteristics, control-tissue selection, tissue composition, RNA-isolation procedures, and normalization strategies; however, these possibilities remain speculative. Importantly, our OSCC group was restricted to early pT1–2N0M0 tumors, whereas many previous cohorts included heterogeneous disease stages.

The marked downregulation of miR-21-5p in dysplastic OL is therefore one of the central findings of the present study. Previous studies of OL have generally assessed miR-21 without consistently distinguishing between its two mature strands. Cervigne et al. reported increased miR-21 expression during progression of selected leukoplakias toward OSCC [35], while Brito et al. described higher miR-21 expression in OL than in normal mucosa and a further increase in OSCC [36]. At first sight, these observations differ from the pronounced miR-21-5p downregulation identified in our cohort. However, direct comparison is limited because earlier studies did not necessarily analyze miR-21-5p and miR-21-3p separately. Increased total miR-21 expression therefore cannot automatically be interpreted as increased miR-21-5p expression.

This distinction may be biologically relevant. Although miR-21-5p and miR-21-3p originate from the same precursor, their different nucleotide sequences allow them to interact with different target transcripts and potentially exert distinct biological effects [30,37,38,39,40,41]. Measurement of total miR-21 may therefore obscure strand-specific expression patterns, supporting separate evaluation of the two mature strands across different stages of oral carcinogenesis.

The marked reduction in miR-21-5p in dysplastic OL, followed by expression in early OSCC that was comparable to healthy controls, is not readily reconciled with the predominant literature reporting increased miR-21/miR-21-5p expression in OSCC and other cancers [32,42,43]. This inconsistency remains unresolved. The biological and methodological explanations discussed here are possible but speculative, and the present cross-sectional study cannot determine whether the observed pattern reflects stage-related biology, tissue composition, normalization or other technical factors, residual confounding, or chance.

Several non-mutually exclusive mechanisms could contribute to the marked reduction in miR-21-5p in OL. Epigenetic regulation of *MIR21* transcription has been demonstrated in other malignancies [44], and changes in Drosha/Dicer processing, Argonaute loading, or strand-specific turnover could alter the balance between the 5p and 3p products of the same precursor [37,40,41]. However, methylation, precursor processing, Argonaute loading, and strand stability were not measured in the present study; these explanations should therefore be regarded as hypotheses requiring direct experimental testing.

Tissue composition and sampling may also influence the observed pattern. Bulk-tissue RT-qPCR reflects miRNA derived from epithelial, stromal, vascular, and inflammatory compartments. In OSCC, miR-21 expression has been localized predominantly to stromal fibroblast-like cells and tumor-associated vessels [45]. Moreover, healthy controls were sampled from buccal mucosa, whereas OL and OSCC specimens arose at several oral sites. Differences in epithelial-to-stromal proportions, inflammatory infiltrates, and anatomical sampling could therefore contribute both to the low OL values and to the apparent return of miR-21-5p toward control levels in early OSCC. The present bulk-tissue design cannot distinguish a cell-intrinsic expression change from a compositional effect.

Normalization is an additional methodological consideration. *SNORD48* was used as the sole endogenous reference, but its stability across healthy oral mucosa, OL, and early OSCC was not independently evaluated against multiple candidate reference genes. Because the 2^−ΔΔCt^ approach assumes a stable normalizer, group-related variation in *SNORD48* could influence the magnitude of the normalized differences. Current qPCR guidance recommends empirical validation of reference genes in the intended biological matrix and, where appropriate, normalization using multiple stable references [46,47]. Accordingly, the present expression estimates warrant confirmation using validated reference genes and, ideally, compartment-resolved analyses.

Importantly, reduced miR-21-5p expression in OL does not constitute evidence of a tumor-suppressive function. miR-21-5p has been reported to repress tumor-suppressive or invasion-regulatory targets including PDCD4, PTEN, RECK, and TIMP3 [27,28,29,42,43,48]. Lower miR-21-5p abundance could theoretically relieve repression of these targets; however, target mRNA and protein expression and downstream pathway activity were not assessed here. The functional consequence of the observed expression pattern therefore cannot be inferred from the present data.

A particularly relevant finding was the association between miR-21-5p expression and epithelial dysplasia grade in patients with OL. Spearman analysis showed a significant inverse correlation between dysplasia grade and miR-21-5p expression (ρ = −0.54; *p* = 0.013), and linear regression likewise demonstrated a significant inverse association (unstandardized regression coefficient B = −0.646; 95% CI −1.162 to −0.131; *p* = 0.017). Under the coding system used in the analysis, lower dysplasia grades were associated with higher miR-21-5p levels than higher grades. Given the small sample size and absence of longitudinal follow-up, this finding should not be interpreted as evidence of prognostic value, but rather as an indication that miR-21-5p expression may be related to biological differences across histopathological grades of OL. Previous studies of total miR-21 have also reported associations with selected histopathological features of dysplasia [36], while our findings specifically highlight miR-21-5p.

Although miR-21-5p and miR-21-3p arise from the same precursor, arm selection and mature-strand stability can differ, and the two strands have distinct target repertoires [37,40,41]. The strong positive correlation observed within the OL group (ρ = 0.71, *p* < 0.001) indicates that participants with relatively higher 3p expression also tended to have relatively higher 5p expression within that group; it does not imply that the two strands had similar mean expression across groups. A moderate positive correlation was also present in healthy controls (ρ = 0.50, *p* = 0.025), whereas no correlation was detected in early OSCC (ρ = −0.04, *p* = 0.880). This loss of within-sample coupling in OSCC may reflect malignant heterogeneity, altered strand selection or processing, or differences in tissue composition, but these interpretations remain hypothesis-generating.

A strength of this study is the simultaneous analysis of clearly defined, histopathologically verified groups. Several limitations should nevertheless be acknowledged. Each group included only 20 participants, reducing statistical power—particularly for miR-21-3p, whose overall *p* value was 0.051—and limiting the precision of subgroup estimates. This single-center, cross-sectional study lacked longitudinal follow-up and therefore cannot determine malignant transformation or establish predictive or prognostic value. The OSCC cohort included only early pT1–2N0M0 tumors, so the findings should not be generalized to advanced or node-positive disease. Controls were deliberately exposure-free and all control specimens were taken from buccal mucosa, whereas tobacco and alcohol exposure occurred in the OL and OSCC groups and lesion sites varied; residual confounding and site-related tissue heterogeneity are therefore possible. Molecular limitations include use of *SNORD48* as a single, non-independently validated reference and analysis of bulk rather than compartment-resolved tissue. Larger multicenter prospective studies with exposure-matched controls, site-aware sampling, validated reference genes, independent replication, and long-term follow-up are required. An additional important limitation is that the unexpected miR-21-5p pattern remains unexplained; without longitudinal or mechanistic validation, this study cannot determine the reason for its divergence from most previous reports.

Taken together, our results show group-specific differences in the expression of miR-21-5p and miR-21-3p. The marked reduction in miR-21-5p in dysplastic OL, followed by levels in early OSCC that were comparable to healthy controls, remains an unresolved inconsistency with most published reports of miR-21/miR-21-5p upregulation. These data do not establish a stage-specific or temporal regulatory model, and the proposed biological and methodological explanations should be considered hypothesis-generating. Separate analysis of the two mature strands may be informative, but their clinical and prognostic utility requires confirmation in larger, independent, longitudinal cohorts [49,50,51].

## 5. Conclusions

This study identified distinct strand-specific expression differences across the three cross-sectional groups. miR-21-5p was markedly downregulated in oral leukoplakia with epithelial dysplasia compared with both healthy oral mucosa and early OSCC, and its expression was significantly associated with epithelial dysplasia grade. In contrast, miR-21-3p showed a different expression pattern, with numerically higher levels in early OSCC, although the between-group difference did not reach statistical significance.

The unexpected miR-21-5p pattern remains unexplained and is inconsistent with much of the published literature. The proposed biological and methodological explanations are speculative, and these data do not establish a stage-specific or sequential molecular trajectory from healthy mucosa through dysplasia to invasive carcinoma.

The present cross-sectional data neither establish prognostic or predictive value nor show that either strand can identify malignant transformation. Confirmation requires larger, independent, longitudinal cohorts with appropriate exposure and sampling-site control.

## Figures and Tables

**Figure 1:**
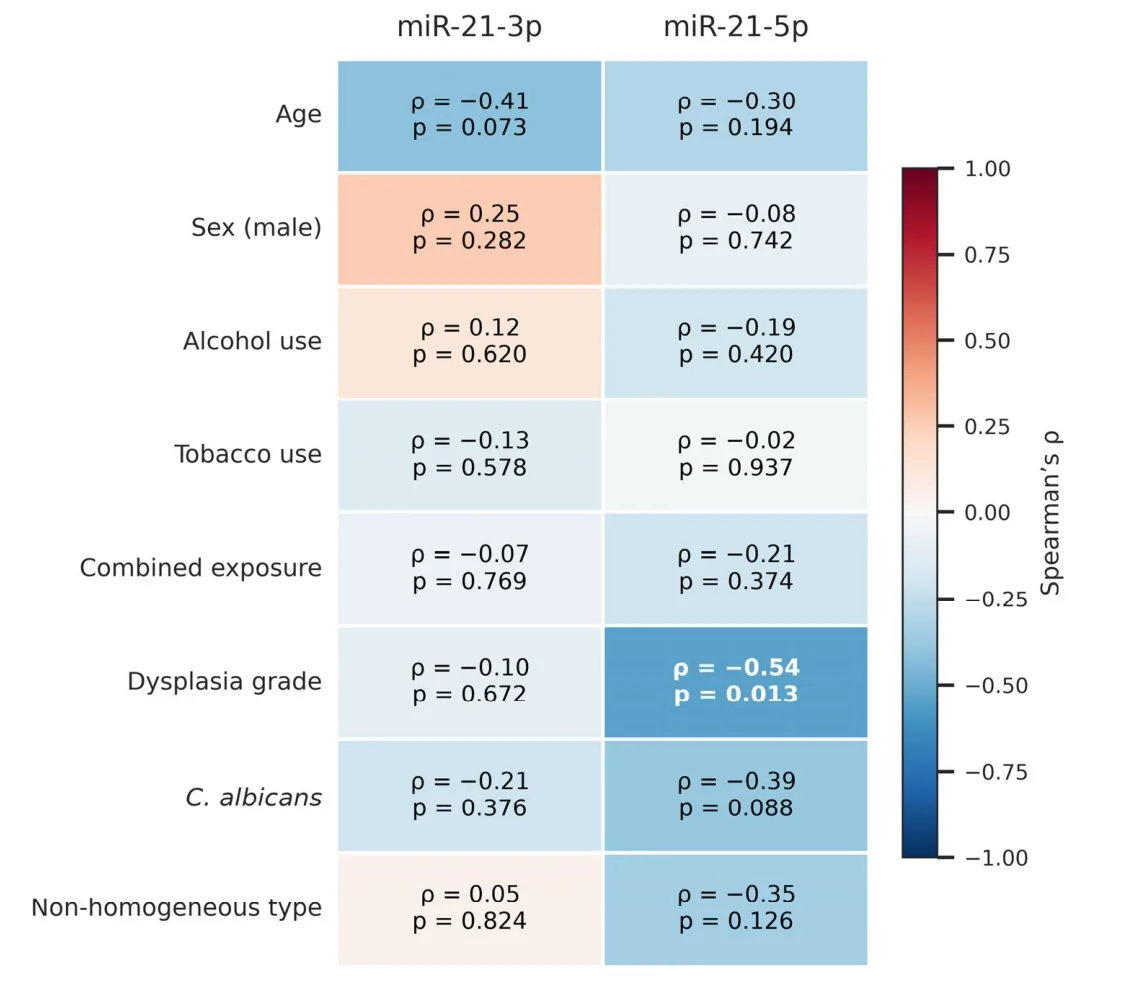
Spearman correlations between clinicopathological variables and miR-21-3p and miR-21-5p expression within the oral leukoplakia group. Cells show Spearman’s ρ and two-sided *p* values. Binary variables were coded as specified in the statistical methods; dysplasia grade was coded from low to high.

**Figure 2:**
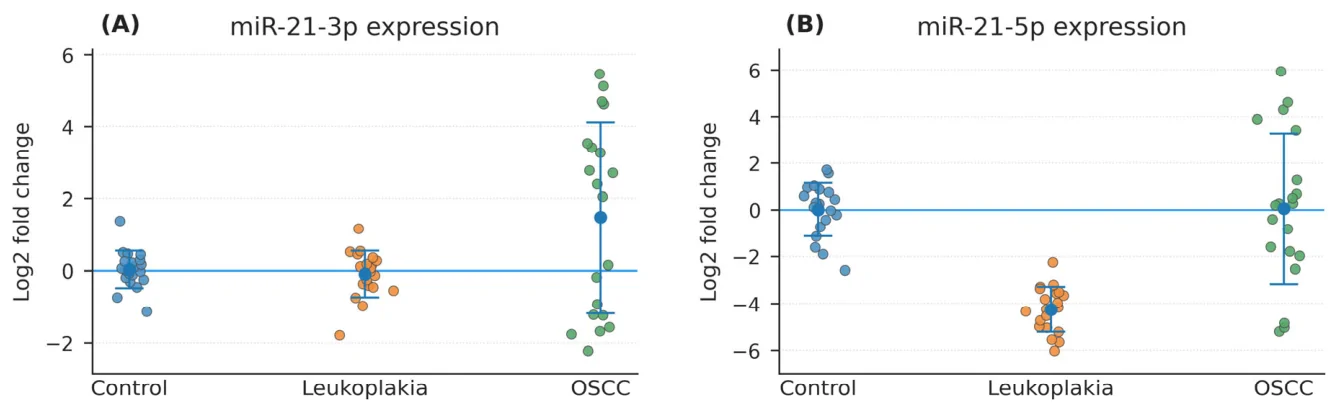
Relative expression of miR-21-3p (**A**) and miR-21-5p (**B**) in healthy oral mucosa, oral leukoplakia, and early oral squamous cell carcinoma. Data are presented as mean log_2_ fold change (log_2_FC) ± SD.

**Figure 3:**
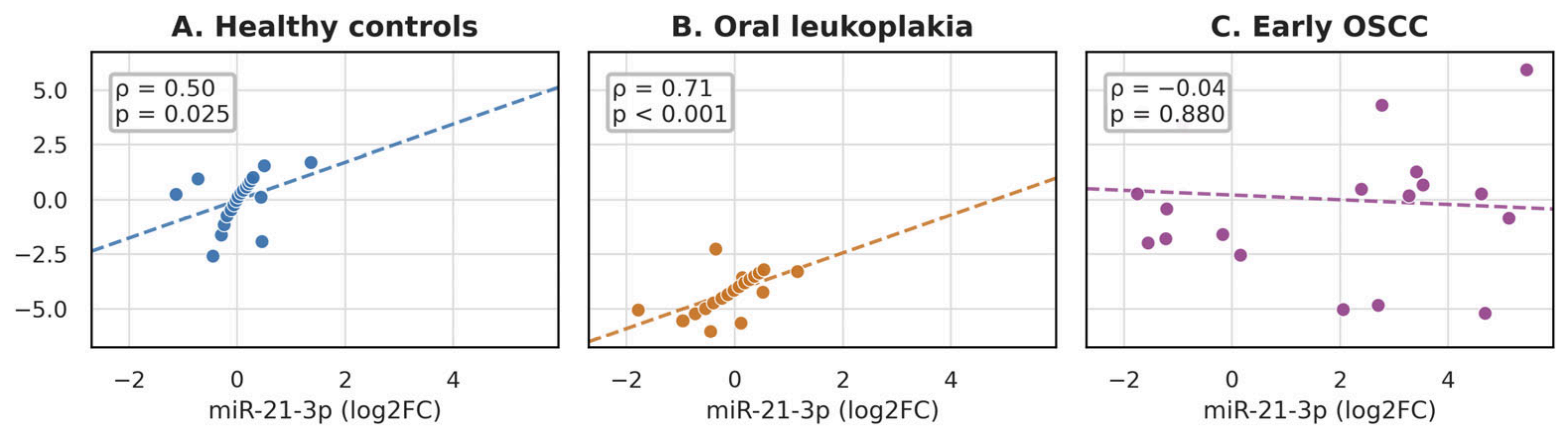
Relationship between miR-21-3p and miR-21-5p expression within healthy controls (**A**), oral leukoplakia (**B**), and early oral squamous cell carcinoma (**C**). Each point represents one participant. Spearman’s rank correlation coefficients (ρ) and two-sided *p* values are shown; dashed lines are least-squares visual guides only.

**Table 1:** Commercially available miRCURY LNA miRNA PCR assays used in this study.

Target	Catalog No.	Product No.
hsa-miR-21-5p	YP00204230	339306
hsa-miR-21-3p	YP00204302	339306
*SNORD48* (endogenous reference gene)	YP00203903	339306

**Table 2:** Demographic, exposure, sampling-site, and clinicopathological characteristics of the study groups (*n* = 20 per group).

Characteristic	Category	Healthy Controls	Oral Leukoplakia	Early OSCC
Age (years)	Mean ± SD (range)	55.5 ± 10.21 (39–72)	60.1 ± 11.93 (37–76)	62.5 ± 11.23 (42–78)
Sex	Male	11 (55)	11 (55)	13 (65)
	Female	9 (45)	9 (45)	7 (35)
Smoking	Yes	0 (0)	6 (30)	13 (65)
	No	20 (100)	14 (70)	7 (35)
Alcohol consumption	Yes	0 (0)	13 (65)	8 (40)
	No	20 (100)	7 (35)	12 (60)
Combined smoking and alcohol	Yes	0 (0)	5 (25)	8 (40)
	No	20 (100)	15 (75)	12 (60)
Sampling/lesion site	Buccal mucosa	20 (100)	4 (20)	0 (0)
	Floor of mouth	0 (0)	8 (40)	3 (15)
	Ventral tongue	0 (0)	6 (30)	0 (0)
	Tongue, not otherwise specified	0 (0)	0 (0)	12 (60)
	Alveolar ridge	0 (0)	2 (10)	5 (25) †
Epithelial dysplasia grade	Low	—	5 (25)	—
	Intermediate	—	8 (40)	—
	High	—	7 (35)	—
*C. albicans* colonization	Present	—	13 (65)	—
	Absent	—	7 (35)	—
Clinical type of leukoplakia	Homogeneous	—	8 (40)	—
	Non-homogeneous	—	12 (60)	—

Note: Values are *n* (%) unless otherwise indicated. Age is reported in years at specimen collection. All healthy controls were non-smokers, did not consume alcohol, and were sampled from buccal mucosa. The OSCC cohort comprised only pT1–2N0M0 tumors. OL-specific variables were not applicable to controls or OSCC. Blue shading identifies the column headings only and does not indicate statistical significance. † All OSCC lesions categorized as alveolar-ridge lesions arose from the mandibular alveolar ridge.

**Table 3:** Association between epithelial dysplasia grade and miR-21-5p expression in oral leukoplakia.

Variable	B	95% CI	*p* Value
Epithelial dysplasia grade	−0.646	−1.162 to −0.131	0.017

B, unstandardized regression coefficient; CI, confidence interval.

**Table 4:** Relative expression of miR-21-3p and miR-21-5p across the study groups.

Variable	Healthy Controls (Mean ± SD)	Oral Leukoplakia (Mean ± SD)	Early OSCC (Mean ± SD)	Overall *p*
**miR-21-3p (log_2_FC)**	0.03 ± 0.51	−0.093 ± 0.64	1.47 ± 2.64	0.051
**miR-21-5p (log_2_FC)**	0.004 ± 1.13	−4.25 ± 0.97	0.06 ± 3.22	<0.001

Note: Values are mean ± SD. Bold type identifies the column headings and variable labels only and does not indicate statistical significance. Overall *p* values were obtained using Welch’s ANOVA. For miR-21-5p, the Games–Howell mean difference was −4.25 log_2_FC for OL versus healthy controls (95% CI, −5.07 to −3.44; *p* < 0.001), −4.31 for OL versus early OSCC (95% CI, −6.19 to −2.42; *p* < 0.001), and 0.06 for early OSCC versus healthy controls (95% CI, −1.85 to 1.96; *p* = 0.997). No post hoc comparisons were interpreted for miR-21-3p because the overall test was not significant (*p* = 0.051).

## Data Availability

The raw data supporting the conclusions of this article will be made available by the authors on request.
